# Bis[μ-1,2-bis­(diphenyl­phosphino)methane-κ^2^ *P*:*P*′]bis­[(η^2^-ethene)nickel(0)] toluene disolvate

**DOI:** 10.1107/S1600536808002110

**Published:** 2008-01-25

**Authors:** Jens Langer, Reinhald Fischer, Helmar Görls, Dirk Walther

**Affiliations:** a Friedrich-Schiller-Universität Jena, Institut für Anorganische und Analytische Chemie, August-Bebel-Strasse 2, D-07743 Jena, Germany

## Abstract

In the title compound, [Ni_2_(C_2_H_4_)_2_(C_25_H_22_P_2_)_2_]·2C_7_H_8_, each Ni atom is coordinated in a trigonal-planar geometry by two P atoms of the bridging 1,2-bis­(diphenyl­phosphino)methane (dppm) ligands and by the centroid of the double bond of an ethene ligand. An eight-membered ring comprising the two Ni atoms, four P atoms and the CH_2_ groups of the two dppm ligands is thus formed. The methyl group in one of the solvent toluene mol­ecules is disordered over two positions with equal occupancies.

## Related literature

For related literature, see: Aresta & Dibenedetto (2007 ▶); Cheng *et al.* (1971 ▶); Fischer *et al.* (2006 ▶); Hoberg *et al.* (1987 ▶); Krüger & Tsay (1972 ▶); Langer *et al.* (2007 ▶); Papai *et al.* (2004 ▶); Wilke & Herrmann (1962 ▶).
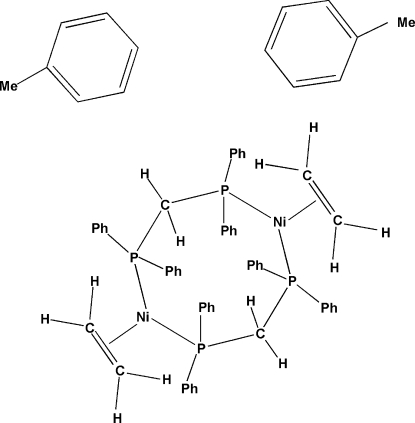


## Experimental

### 

#### Crystal data

[Ni_2_(C_2_H_4_)_2_(C_25_H_22_P_2_)_2_]·2C_7_H_8_
*M* *_r_* = 1126.52
Triclinic, 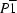

*a* = 13.0963 (12) Å
*b* = 15.2367 (16) Å
*c* = 15.6177 (16) Å
α = 70.566 (4)°
β = 78.727 (4)°
γ = 78.689 (7)°
*V* = 2853.1 (5) Å^3^
*Z* = 2
Mo *K*α radiation
μ = 0.81 mm^−1^
*T* = 183 (2) K
0.05 × 0.05 × 0.03 mm

#### Data collection

Nonius KappaCCD diffractometer
Absorption correction: multi-scan (Blessing, 1997 ▶) *T*_min_ = 0.834, *T*_max_ = 0.994
16239 measured reflections
11467 independent reflections
8057 reflections with *I* > 2σ(*I*)
*R*_int_ = 0.050

#### Refinement

*R*[*F* ^2^ > 2σ(*F* ^2^)] = 0.086
*wR*(*F* ^2^) = 0.233
*S* = 1.14
11467 reflections
666 parameters
H-atom parameters constrained
Δρ_max_ = 1.46 e Å^−3^
Δρ_min_ = −0.63 e Å^−3^

### 

Data collection: *COLLECT* (Nonius, 1998 ▶); cell refinement: *DENZO* (Otwinowski & Minor, 1997 ▶); data reduction: *DENZO*; program(s) used to solve structure: *SHELXS97* (Sheldrick, 2008 ▶); program(s) used to refine structure: *SHELXL97* (Sheldrick, 2008 ▶); molecular graphics: *SHELXTL/PC* (Sheldrick, 2008 ▶); software used to prepare material for publication: *SHELXL97*.

## Supplementary Material

Crystal structure: contains datablocks I, global. DOI: 10.1107/S1600536808002110/fj2093sup1.cif

Structure factors: contains datablocks I. DOI: 10.1107/S1600536808002110/fj2093Isup2.hkl

Additional supplementary materials:  crystallographic information; 3D view; checkCIF report

## Figures and Tables

**Fig. 1.**
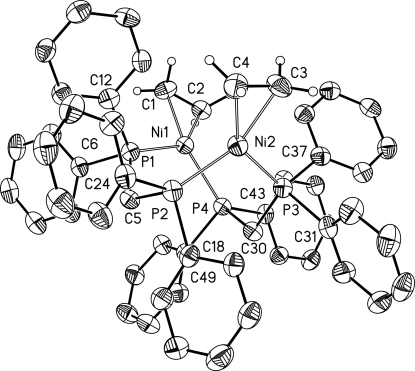
Molecular structure of 1. Displacement ellipsoids are drawn at the 40% probability level. Except for the ethene ligands, all H atoms have been omitted for clarity.

## supplementary crystallographic information

### Comment

Our recent research has shown, that 1,2-bis(diphenylphosphino)methane (dppm) is a suitable ligand to promote \&s-hydride elimination in nickelacyclic carboxylates under formation of nickel acrylate complexes (Fischer *et al.* 2006, Langer *et al.* 2007). This reaction models the final step of the hypothetical acrylic acid synthesis from ethene and CO_2_, catalyzed by homogeneous Ni catalysts (Hoberg *et al.* 1987; Papai & Aresta 2004, Langer *et al.* 2007, Aresta & Dibenedetto 2007). In connection with these investigations we were also interested in isolating a well defined dppm Ni complex containing ethene as a ligand, which can be considered as starting complex for the first step of this catalytic reaction. Since the method of choice for preparing nickel ethene complexes is often the reduction of Ni(acac)_2_ with Al(Et)_3_ in presence of ligands (Wilke & Herrmann 1962) we used this method. In the presence of dppm a mixture of products were formed as judged by the ^31^P NMR spectrum of the reaction solution. After removing of half of the solvent in vacuum, subsequent cooling, filtration and storing the mother liquor of the reaction at -40 °C orange crystals of the desired ethene nickel(0) complex having the composition {(µ-dppm)_2_[Ni(C_2_H_4_)]_2_(toluene)_2_} were isolated together with [(dppm)Ni(et)(acac)(toluene)] as by-product. The crystal structure of this new Ni(0) complex, presented in Figure 1, shows that a nickel(0) dimer is formed in which two µ-dppm bridges connect the two Ni atoms, thus forming a eight-membered inner ring of the two Ni atoms, four P atoms and the two CH_2_ groups of the dppm ligand. The nickel atoms are in a distorted trigonal planar environment formed by two phosphorous atoms of two dppm ligands and the centroid of the coordinated double bond of ethene. The angle between the planes defined by Ni1P1P4 (Ni2P2P3) and Ni1C1C2 (Ni2C3C4) is 12.27 ° (10.21 °) and lies in typical range for ethene complexes of nickel. The Ni—P bond between 2.1396 (18) and 2.1665 (19) Å and the Ni—C bond length between 1.969 (7) and 1.976 (7) Å compare well with those found in bis(triphenylphosphine)(ethene) nickel (Cheng *et al.* 1971) and the corresponding tricyclohexylphosphine complex (Krüger & Tsay 1972). As expected, the C=C bonds of the coordinated olefins are lengthened compared with the uncoordinated olefin.

### Experimental

All manipulations were carried out by using modified Schlenk techniques under an atmosphere of argon. Prior to use, toluene was distilled over sodium/benzophenone.

A filtered solution of Ni(acac)_2_ (1.06 g, 4.12 mmol) in toluene (10 ml) was treated with 1,2 bis(diphenylphosphino)methane (1.58 g, 4.11 mmol). The resulting green solution was cooled to 0 °C and placed under an atmosphere of ethene. Afterwards AlEt_3_ (1.25 ml, 9.03 mmol) was added dropwise at this temperature with rapid stirring. The resulting brown solution was stirred for an hour at 0 °C and then stored at -20 °C for three days. The formed partial crystalline precipitate was removed by filtration. Afterwards the brown mother liquor was reduced to half of the original volume and stored for three weeks at -40 °C. During this time, well shaped orange crystals of {(µ-dppm)_2_[Ni(C_2_H_4_)]_2_(toluene)_2_} precipitated from the brown oily solution, together with pale yellow crystals of [(dppm)Ni(et)(acac)(toluene)]. After separation, the cystals of{(µ-dppm)_2_[Ni(C_2_H_4_)]_2_(toluene)_2_} so formed were suitable for X-ray diffraction.

### Refinement

The quality of the crystal was very bad (mosaicity 2.512 (3)°), so hundreds of high order reflections are missing from the data set. All hydrogen atoms were set to idealized positions and were refined with 1.2 times (1.5 for methyl groups) the isotropic displacement parameter of the corresponding carbon atom. The central Ni-atom shows an interaction to the ethene molecules, giving the C-atoms a partial *sp*^3^ hybridization. For this reason, the AFIX 2 instruction was used for these hydrogen positions. Atoms C7TB and C7TC of a toluene molecule are disordered over two positions with equal occupancies. The carbon atoms of disordered part of the toluene molecule were refined using isotropic thermal parameters.

### Crystal data

**Table:**

[Ni_2_(C_2_H_4_)_2_(C_25_H_22_P_2_)_2_]·2C_7_H_8_	*Z* = 2
*M**_r_* = 1126.52	*F*_000_ = 1184
Triclinic, *P*1	*D*_x_ = 1.311 Mg m^−^^3^
Hall symbol: -P 1	Mo *K*α radiation λ = 0.71073 Å
*a* = 13.0963 (12) Å	Cell parameters from 16239 reflections
*b* = 15.2367 (16) Å	θ = 2.0–27.5º
*c* = 15.6177 (16) Å	µ = 0.81 mm^−^^1^
α = 70.566 (4)º	*T* = 183 (2) K
β = 78.727 (4)º	Prism, orange
γ = 78.689 (7)º	0.05 × 0.05 × 0.03 mm
*V* = 2853.1 (5) Å^3^	

### Data collection

**Table:**

Nonius KappaCCD diffractometer	11467 independent reflections
Radiation source: fine-focus sealed tube	8057 reflections with *I* > 2σ(*I*)
Monochromator: graphite	*R*_int_ = 0.050
*T* = 183(2) K	θ_max_ = 27.5º
φ and ω scans	θ_min_ = 2.0º
Absorption correction: multi-scan(Blessing, 1997)	*h* = −14→16
*T*_min_ = 0.834, *T*_max_ = 0.994	*k* = −17→19
16239 measured reflections	*l* = −17→20

### Refinement

**Table:**

Refinement on *F*^2^	Secondary atom site location: difference Fourier map
Least-squares matrix: full	Hydrogen site location: inferred from neighbouring sites
*R*[*F*^2^ > 2σ(*F*^2^)] = 0.086	H-atom parameters constrained
*wR*(*F*^2^) = 0.233	*w* = 1/[σ^2^(*F*_o_^2^) + (0.0587*P*)^2^ + 15.5715*P*] where *P* = (*F*_o_^2^ + 2*F*_c_^2^)/3
*S* = 1.14	(Δ/σ)_max_ = 0.001
11467 reflections	Δρ_max_ = 1.46 e Å^−^^3^
666 parameters	Δρ_min_ = −0.63 e Å^−^^3^
Primary atom site location: structure-invariant direct methods	Extinction correction: none

### Special details

**Table:**

Geometry. All e.s.d.'s (except the e.s.d. in the dihedral angle between two l.s. planes) are estimated using the full covariance matrix. The cell e.s.d.'s are taken into account individually in the estimation of e.s.d.'s in distances, angles and torsion angles; correlations between e.s.d.'s in cell parameters are only used when they are defined by crystal symmetry. An approximate (isotropic) treatment of cell e.s.d.'s is used for estimating e.s.d.'s involving l.s. planes.
Refinement. Refinement of *F*^2^ against ALL reflections. The weighted *R*-factor *wR* and goodness of fit *S* are based on *F*^2^, conventional *R*-factors *R* are based on *F*, with *F* set to zero for negative *F*^2^. The threshold expression of *F*^2^ > σ(*F*^2^) is used only for calculating *R*-factors(gt) *etc*. and is not relevant to the choice of reflections for refinement. *R*-factors based on *F*^2^ are statistically about twice as large as those based on *F*, and *R*- factors based on ALL data will be even larger.

### Fractional atomic coordinates and isotropic or equivalent isotropic displacement parameters (Å^2^)

**Table:**

	*x*	*y*	*z*	*U*_iso_*/*U*_eq_	Occ. (<1)
Ni1	0.79325 (6)	0.90928 (6)	0.58612 (5)	0.0336 (2)	
Ni2	0.94947 (7)	0.73710 (6)	0.79352 (6)	0.0375 (2)	
P1	0.73773 (13)	0.94131 (12)	0.71209 (11)	0.0338 (4)	
P2	0.80241 (14)	0.76591 (12)	0.87616 (11)	0.0366 (4)	
P3	0.92014 (13)	0.65602 (12)	0.71245 (11)	0.0355 (4)	
P4	0.74457 (13)	0.77889 (12)	0.59627 (11)	0.0332 (4)	
C1	0.8393 (6)	1.0309 (5)	0.5101 (4)	0.0448 (17)	
H1A	0.7843	1.0867	0.4999	0.054*	
H1B	0.9069	1.0434	0.5198	0.054*	
C2	0.8452 (6)	0.9699 (5)	0.4560 (4)	0.0426 (15)	
H2A	0.9165	0.9444	0.4320	0.051*	
H2B	0.7939	0.9877	0.4121	0.051*	
C3	1.1034 (6)	0.7358 (6)	0.7689 (6)	0.0543 (19)	
H3A	1.1473	0.6734	0.7859	0.065*	
H3B	1.1316	0.7797	0.7103	0.065*	
C4	1.0589 (6)	0.7737 (6)	0.8399 (6)	0.0533 (19)	
H4A	1.0589	0.8419	0.8267	0.064*	
H4B	1.0745	0.7356	0.9022	0.064*	
C5	0.6950 (5)	0.8506 (5)	0.8197 (4)	0.0353 (13)	
H5A	0.6532	0.8827	0.8635	0.042*	
H5B	0.6480	0.8149	0.8060	0.042*	
C6	0.6151 (5)	1.0238 (5)	0.6959 (4)	0.0354 (14)	
C7	0.6130 (6)	1.1210 (5)	0.6618 (5)	0.0458 (16)	
H7A	0.6770	1.1466	0.6482	0.055*	
C8	0.5196 (6)	1.1810 (6)	0.6473 (5)	0.0521 (19)	
H8A	0.5200	1.2470	0.6256	0.063*	
C9	0.4261 (6)	1.1457 (6)	0.6642 (5)	0.0501 (18)	
H9A	0.3623	1.1872	0.6534	0.060*	
C10	0.4258 (6)	1.0502 (6)	0.6966 (5)	0.0492 (18)	
H10A	0.3616	1.0255	0.7082	0.059*	
C11	0.5192 (5)	0.9896 (5)	0.7126 (4)	0.0422 (15)	
H11A	0.5177	0.9237	0.7354	0.051*	
C12	0.8149 (5)	1.0058 (5)	0.7518 (4)	0.0389 (15)	
C13	0.7702 (6)	1.0600 (5)	0.8089 (4)	0.0423 (15)	
H13A	0.6965	1.0658	0.8286	0.051*	
C14	0.8324 (6)	1.1061 (5)	0.8374 (5)	0.0475 (17)	
H14A	0.8011	1.1433	0.8766	0.057*	
C15	0.9390 (6)	1.0980 (5)	0.8091 (5)	0.0492 (18)	
H15A	0.9812	1.1299	0.8285	0.059*	
C16	0.9850 (6)	1.0438 (5)	0.7526 (4)	0.0441 (16)	
H16A	1.0590	1.0375	0.7341	0.053*	
C17	0.9231 (5)	0.9981 (5)	0.7225 (4)	0.0393 (15)	
H17A	0.9545	0.9621	0.6824	0.047*	
C18	0.7414 (6)	0.6594 (5)	0.9333 (4)	0.0393 (15)	
C19	0.6330 (6)	0.6560 (5)	0.9527 (5)	0.0468 (17)	
H19A	0.5853	0.7116	0.9317	0.056*	
C20	0.5944 (7)	0.5725 (6)	1.0020 (6)	0.0556 (19)	
H20A	0.5206	0.5717	1.0148	0.067*	
C21	0.6616 (8)	0.4900 (6)	1.0331 (6)	0.064 (2)	
H21A	0.6341	0.4332	1.0668	0.077*	
C22	0.7696 (7)	0.4909 (5)	1.0145 (5)	0.0535 (19)	
H22A	0.8166	0.4349	1.0358	0.064*	
C23	0.8079 (6)	0.5747 (5)	0.9646 (4)	0.0418 (15)	
H23A	0.8819	0.5747	0.9511	0.050*	
C24	0.7971 (5)	0.8071 (5)	0.9760 (4)	0.0411 (15)	
C25	0.7317 (6)	0.7762 (6)	1.0571 (5)	0.0531 (19)	
H25A	0.6868	0.7319	1.0629	0.064*	
C26	0.7302 (7)	0.8088 (7)	1.1312 (6)	0.063 (2)	
H26A	0.6864	0.7852	1.1874	0.075*	
C27	0.7924 (7)	0.8752 (6)	1.1226 (5)	0.058 (2)	
H27A	0.7906	0.8983	1.1724	0.069*	
C28	0.8570 (7)	0.9080 (6)	1.0419 (5)	0.0519 (19)	
H28A	0.8988	0.9547	1.0357	0.062*	
C29	0.8616 (6)	0.8734 (5)	0.9688 (5)	0.0444 (16)	
H29A	0.9085	0.8948	0.9141	0.053*	
C30	0.7847 (5)	0.6702 (5)	0.6872 (4)	0.0373 (14)	
H30A	0.7353	0.6689	0.7443	0.045*	
H30B	0.7775	0.6155	0.6691	0.045*	
C31	0.9419 (5)	0.5287 (5)	0.7717 (4)	0.0371 (14)	
C32	0.8869 (6)	0.4623 (5)	0.7658 (5)	0.0495 (18)	
H32A	0.8331	0.4825	0.7276	0.059*	
C33	0.9073 (7)	0.3675 (5)	0.8135 (6)	0.059 (2)	
H33A	0.8673	0.3241	0.8086	0.071*	
C34	0.9861 (7)	0.3367 (5)	0.8680 (5)	0.057 (2)	
H34A	1.0011	0.2720	0.9010	0.068*	
C35	1.0417 (7)	0.4000 (6)	0.8740 (6)	0.062 (2)	
H35A	1.0963	0.3788	0.9113	0.074*	
C36	1.0215 (6)	0.4940 (6)	0.8275 (5)	0.0531 (19)	
H36A	1.0623	0.5364	0.8334	0.064*	
C37	1.0022 (5)	0.6633 (5)	0.6006 (4)	0.0388 (14)	
C38	1.0313 (5)	0.5889 (5)	0.5651 (4)	0.0411 (15)	
H38A	1.0081	0.5302	0.5991	0.049*	
C39	1.0940 (6)	0.5983 (5)	0.4808 (5)	0.0477 (17)	
H39A	1.1134	0.5464	0.4574	0.057*	
C40	1.1282 (5)	0.6839 (5)	0.4307 (5)	0.0458 (17)	
H40A	1.1706	0.6907	0.3727	0.055*	
C41	1.1006 (6)	0.7593 (5)	0.4651 (5)	0.0439 (16)	
H41A	1.1240	0.8179	0.4309	0.053*	
C42	1.0384 (5)	0.7491 (5)	0.5503 (5)	0.0423 (15)	
H42A	1.0204	0.8007	0.5743	0.051*	
C43	0.7689 (5)	0.7306 (5)	0.4990 (4)	0.0359 (14)	
C44	0.8451 (5)	0.7592 (5)	0.4255 (4)	0.0401 (15)	
H44A	0.8838	0.8066	0.4235	0.048*	
C45	0.8665 (6)	0.7198 (5)	0.3537 (5)	0.0450 (16)	
H45A	0.9196	0.7401	0.3036	0.054*	
C46	0.8102 (6)	0.6512 (5)	0.3558 (5)	0.0449 (16)	
H46A	0.8243	0.6245	0.3070	0.054*	
C47	0.7335 (6)	0.6215 (5)	0.4289 (5)	0.0472 (17)	
H47A	0.6955	0.5737	0.4308	0.057*	
C48	0.7116 (6)	0.6613 (5)	0.4999 (5)	0.0418 (15)	
H48A	0.6576	0.6415	0.5493	0.050*	
C49	0.6002 (5)	0.7883 (4)	0.6211 (4)	0.0343 (13)	
C50	0.5452 (5)	0.8611 (5)	0.5580 (5)	0.0425 (15)	
H50A	0.5831	0.9012	0.5065	0.051*	
C51	0.4361 (5)	0.8758 (5)	0.5694 (5)	0.0478 (17)	
H51A	0.4002	0.9251	0.5254	0.057*	
C52	0.3800 (6)	0.8198 (6)	0.6436 (6)	0.0554 (19)	
H52A	0.3053	0.8306	0.6516	0.066*	
C53	0.4324 (6)	0.7472 (6)	0.7073 (6)	0.057 (2)	
H53A	0.3935	0.7082	0.7590	0.069*	
C54	0.5423 (5)	0.7312 (5)	0.6957 (5)	0.0447 (16)	
H54A	0.5777	0.6808	0.7393	0.054*	
C1TA	0.5103 (7)	0.5123 (7)	0.3440 (7)	0.066 (2)	
C2TA	0.5831 (8)	0.5413 (8)	0.2679 (7)	0.080 (3)	
H2TA	0.5618	0.5904	0.2158	0.096*	
C3TA	0.6876 (8)	0.4994 (10)	0.2664 (8)	0.092 (4)	
H3TA	0.7377	0.5210	0.2140	0.111*	
C4TA	0.7178 (8)	0.4275 (9)	0.3399 (8)	0.082 (3)	
H4TA	0.7890	0.3985	0.3384	0.099*	
C5TA	0.6461 (8)	0.3962 (7)	0.4165 (8)	0.076 (3)	
H5TA	0.6670	0.3457	0.4679	0.091*	
C6TA	0.5430 (8)	0.4400 (7)	0.4170 (7)	0.070 (2)	
H6TA	0.4933	0.4191	0.4699	0.084*	
C7TA	0.3960 (7)	0.5584 (8)	0.3462 (8)	0.080 (3)	
H7TA	0.3560	0.5289	0.4054	0.120*	
H7TB	0.3662	0.5504	0.2968	0.120*	
H7TC	0.3924	0.6256	0.3376	0.120*	
C1TB	0.3093 (12)	0.7418 (12)	0.0031 (15)	0.121 (6)	
H1TA	0.2669	0.7046	−0.0091	0.146*	
C2TB	0.3580 (14)	0.7099 (10)	0.0797 (14)	0.125 (6)	
H2TB	0.3487	0.6503	0.1231	0.150*	
C3TB	0.4189 (10)	0.7641 (11)	0.0926 (10)	0.106 (4)	
H3TB	0.4545	0.7400	0.1449	0.127*	
C4TB	0.4329 (11)	0.8513 (10)	0.0356 (10)	0.098 (4)	
H4TB	0.4733	0.8888	0.0496	0.118*	0.50
C5TB	0.3894 (10)	0.8821 (9)	−0.0393 (10)	0.094 (4)	
H5TB	0.4031	0.9409	−0.0825	0.112*	
C6TB	0.3244 (10)	0.8322 (10)	−0.0573 (9)	0.095 (4)	
H6TB	0.2897	0.8582	−0.1101	0.114*	0.50
C7TB	0.2704 (19)	0.8666 (17)	−0.1299 (16)	0.093 (7)*	0.50
H7TD	0.2881	0.9291	−0.1664	0.139*	0.50
H7TE	0.1947	0.8712	−0.1079	0.139*	0.50
H7TF	0.2892	0.8244	−0.1678	0.139*	0.50
C7TC	0.486 (3)	0.923 (2)	0.039 (2)	0.140 (11)*	0.50
H7TG	0.4795	0.9777	−0.0157	0.210*	0.50
H7TH	0.5610	0.8991	0.0428	0.210*	0.50
H7TI	0.4551	0.9427	0.0939	0.210*	0.50

### Atomic displacement parameters (Å^2^)

**Table:**

	*U*^11^	*U*^22^	*U*^33^	*U*^12^	*U*^13^	*U*^23^
Ni1	0.0344 (4)	0.0384 (5)	0.0293 (4)	−0.0054 (3)	−0.0067 (3)	−0.0105 (3)
Ni2	0.0354 (5)	0.0443 (5)	0.0360 (4)	−0.0035 (4)	−0.0094 (3)	−0.0154 (4)
P1	0.0339 (8)	0.0384 (9)	0.0293 (8)	−0.0028 (7)	−0.0070 (6)	−0.0107 (6)
P2	0.0383 (9)	0.0411 (9)	0.0313 (8)	−0.0021 (7)	−0.0101 (7)	−0.0113 (7)
P3	0.0362 (9)	0.0383 (9)	0.0331 (8)	−0.0030 (7)	−0.0104 (7)	−0.0107 (7)
P4	0.0323 (8)	0.0379 (9)	0.0298 (8)	−0.0048 (6)	−0.0072 (6)	−0.0091 (6)
C1	0.057 (4)	0.038 (4)	0.038 (4)	−0.014 (3)	−0.017 (3)	0.000 (3)
C2	0.041 (4)	0.046 (4)	0.037 (3)	−0.005 (3)	−0.008 (3)	−0.006 (3)
C3	0.035 (4)	0.079 (6)	0.058 (5)	−0.012 (4)	−0.006 (3)	−0.031 (4)
C4	0.047 (4)	0.059 (5)	0.065 (5)	−0.001 (3)	−0.024 (4)	−0.028 (4)
C5	0.031 (3)	0.043 (4)	0.031 (3)	−0.005 (3)	−0.006 (2)	−0.010 (3)
C6	0.035 (3)	0.047 (4)	0.027 (3)	0.000 (3)	−0.009 (2)	−0.016 (3)
C7	0.047 (4)	0.050 (4)	0.042 (4)	−0.002 (3)	−0.011 (3)	−0.016 (3)
C8	0.064 (5)	0.047 (4)	0.041 (4)	0.016 (4)	−0.017 (3)	−0.015 (3)
C9	0.047 (4)	0.063 (5)	0.038 (4)	0.013 (4)	−0.015 (3)	−0.019 (3)
C10	0.039 (4)	0.073 (5)	0.036 (4)	−0.001 (3)	−0.008 (3)	−0.018 (3)
C11	0.042 (4)	0.051 (4)	0.033 (3)	−0.003 (3)	−0.006 (3)	−0.014 (3)
C12	0.044 (4)	0.037 (3)	0.033 (3)	−0.005 (3)	−0.013 (3)	−0.004 (3)
C13	0.044 (4)	0.051 (4)	0.037 (3)	−0.004 (3)	−0.006 (3)	−0.021 (3)
C14	0.053 (5)	0.048 (4)	0.048 (4)	−0.004 (3)	−0.010 (3)	−0.023 (3)
C15	0.061 (5)	0.049 (4)	0.042 (4)	−0.013 (4)	−0.016 (3)	−0.013 (3)
C16	0.039 (4)	0.066 (5)	0.031 (3)	−0.018 (3)	−0.009 (3)	−0.012 (3)
C17	0.045 (4)	0.043 (4)	0.029 (3)	0.000 (3)	−0.007 (3)	−0.012 (3)
C18	0.048 (4)	0.037 (4)	0.034 (3)	−0.003 (3)	−0.007 (3)	−0.013 (3)
C19	0.048 (4)	0.047 (4)	0.046 (4)	−0.007 (3)	−0.008 (3)	−0.015 (3)
C20	0.054 (5)	0.053 (5)	0.062 (5)	−0.013 (4)	−0.005 (4)	−0.020 (4)
C21	0.093 (7)	0.045 (5)	0.053 (5)	−0.018 (4)	−0.003 (4)	−0.012 (4)
C22	0.068 (5)	0.039 (4)	0.050 (4)	0.001 (4)	−0.014 (4)	−0.011 (3)
C23	0.047 (4)	0.043 (4)	0.035 (3)	0.000 (3)	−0.008 (3)	−0.014 (3)
C24	0.039 (4)	0.048 (4)	0.037 (3)	0.004 (3)	−0.012 (3)	−0.015 (3)
C25	0.042 (4)	0.076 (6)	0.046 (4)	−0.005 (4)	−0.004 (3)	−0.028 (4)
C26	0.061 (5)	0.092 (7)	0.044 (4)	−0.012 (5)	−0.004 (4)	−0.033 (4)
C27	0.063 (5)	0.074 (6)	0.043 (4)	0.007 (4)	−0.018 (4)	−0.031 (4)
C28	0.064 (5)	0.052 (4)	0.046 (4)	0.001 (4)	−0.024 (4)	−0.019 (3)
C29	0.054 (4)	0.046 (4)	0.034 (3)	−0.007 (3)	−0.014 (3)	−0.010 (3)
C30	0.034 (3)	0.044 (4)	0.033 (3)	−0.004 (3)	−0.009 (3)	−0.009 (3)
C31	0.038 (3)	0.045 (4)	0.028 (3)	0.001 (3)	−0.006 (3)	−0.014 (3)
C32	0.049 (4)	0.046 (4)	0.052 (4)	−0.007 (3)	−0.015 (3)	−0.009 (3)
C33	0.058 (5)	0.041 (4)	0.072 (5)	−0.009 (4)	−0.007 (4)	−0.007 (4)
C34	0.079 (6)	0.037 (4)	0.046 (4)	0.005 (4)	−0.014 (4)	−0.005 (3)
C35	0.071 (6)	0.055 (5)	0.060 (5)	0.016 (4)	−0.036 (4)	−0.017 (4)
C36	0.061 (5)	0.050 (4)	0.051 (4)	0.001 (4)	−0.028 (4)	−0.014 (3)
C37	0.034 (3)	0.044 (4)	0.040 (3)	−0.005 (3)	−0.013 (3)	−0.011 (3)
C38	0.044 (4)	0.045 (4)	0.035 (3)	−0.005 (3)	−0.007 (3)	−0.012 (3)
C39	0.050 (4)	0.051 (4)	0.045 (4)	−0.002 (3)	−0.008 (3)	−0.021 (3)
C40	0.036 (4)	0.062 (5)	0.039 (4)	−0.010 (3)	−0.005 (3)	−0.014 (3)
C41	0.044 (4)	0.045 (4)	0.043 (4)	−0.010 (3)	−0.008 (3)	−0.009 (3)
C42	0.040 (4)	0.044 (4)	0.046 (4)	−0.007 (3)	−0.012 (3)	−0.014 (3)
C43	0.039 (3)	0.042 (4)	0.029 (3)	0.002 (3)	−0.011 (3)	−0.015 (3)
C44	0.039 (4)	0.047 (4)	0.036 (3)	−0.008 (3)	−0.007 (3)	−0.014 (3)
C45	0.048 (4)	0.051 (4)	0.036 (3)	−0.005 (3)	−0.004 (3)	−0.016 (3)
C46	0.049 (4)	0.054 (4)	0.038 (4)	0.002 (3)	−0.010 (3)	−0.024 (3)
C47	0.061 (5)	0.045 (4)	0.040 (4)	−0.006 (3)	−0.010 (3)	−0.018 (3)
C48	0.043 (4)	0.041 (4)	0.043 (4)	−0.007 (3)	−0.008 (3)	−0.013 (3)
C49	0.036 (3)	0.039 (3)	0.034 (3)	−0.006 (3)	−0.008 (3)	−0.017 (3)
C50	0.039 (4)	0.052 (4)	0.036 (3)	−0.002 (3)	−0.008 (3)	−0.015 (3)
C51	0.032 (4)	0.054 (4)	0.059 (4)	0.003 (3)	−0.019 (3)	−0.018 (4)
C52	0.034 (4)	0.068 (5)	0.062 (5)	−0.006 (3)	−0.009 (3)	−0.017 (4)
C53	0.042 (4)	0.069 (5)	0.056 (5)	−0.016 (4)	0.004 (3)	−0.015 (4)
C54	0.037 (4)	0.044 (4)	0.050 (4)	−0.005 (3)	−0.013 (3)	−0.008 (3)
C1TA	0.042 (5)	0.071 (6)	0.090 (7)	−0.002 (4)	−0.016 (4)	−0.031 (5)
C2TA	0.053 (6)	0.111 (9)	0.078 (7)	−0.001 (5)	−0.013 (5)	−0.036 (6)
C3TA	0.060 (6)	0.149 (11)	0.083 (7)	−0.006 (7)	−0.008 (5)	−0.061 (8)
C4TA	0.052 (5)	0.125 (9)	0.099 (8)	0.012 (6)	−0.017 (6)	−0.083 (7)
C5TA	0.062 (6)	0.084 (7)	0.102 (8)	0.009 (5)	−0.035 (6)	−0.052 (6)
C6TA	0.064 (6)	0.072 (6)	0.079 (6)	−0.019 (5)	−0.012 (5)	−0.024 (5)
C7TA	0.051 (5)	0.086 (7)	0.091 (7)	−0.003 (5)	−0.005 (5)	−0.018 (6)
C1TB	0.086 (10)	0.097 (11)	0.207 (19)	−0.025 (8)	0.027 (11)	−0.101 (13)
C2TB	0.099 (12)	0.066 (8)	0.177 (17)	−0.016 (8)	0.068 (11)	−0.041 (10)
C3TB	0.065 (7)	0.119 (11)	0.106 (10)	0.010 (7)	0.017 (6)	−0.029 (8)
C4TB	0.097 (9)	0.103 (10)	0.098 (9)	−0.030 (7)	0.034 (8)	−0.053 (8)
C5TB	0.080 (8)	0.084 (8)	0.112 (10)	−0.027 (6)	0.046 (7)	−0.047 (7)
C6TB	0.082 (8)	0.110 (10)	0.091 (8)	0.004 (7)	0.010 (6)	−0.052 (7)

### Geometric parameters (Å, °)

**Table:**

Ni1—C2	1.976 (7)	C31—C32	1.387 (10)
Ni1—C1	1.976 (6)	C32—C33	1.387 (10)
Ni1—P1	2.1396 (18)	C32—H32A	0.9500
Ni1—P4	2.1508 (19)	C33—C34	1.378 (11)
Ni2—C4	1.969 (7)	C33—H33A	0.9500
Ni2—C3	1.974 (7)	C34—C35	1.353 (12)
Ni2—P2	2.154 (2)	C34—H34A	0.9500
Ni2—P3	2.1665 (19)	C35—C36	1.372 (11)
P1—C6	1.838 (6)	C35—H35A	0.9500
P1—C12	1.847 (7)	C36—H36A	0.9500
P1—C5	1.854 (6)	C37—C38	1.380 (10)
P2—C18	1.826 (7)	C37—C42	1.402 (9)
P2—C24	1.849 (7)	C38—C39	1.389 (10)
P2—C5	1.854 (6)	C38—H38A	0.9500
P3—C31	1.842 (7)	C39—C40	1.388 (10)
P3—C37	1.844 (7)	C39—H39A	0.9500
P3—C30	1.848 (6)	C40—C41	1.382 (10)
P4—C49	1.841 (7)	C40—H40A	0.9500
P4—C43	1.849 (6)	C41—C42	1.395 (10)
P4—C30	1.850 (6)	C41—H41A	0.9500
C1—C2	1.433 (10)	C42—H42A	0.9500
C1—H1A	0.9900	C43—C44	1.376 (9)
C1—H1B	0.9900	C43—C48	1.404 (9)
C2—H2A	0.9900	C44—C45	1.400 (9)
C2—H2B	0.9900	C44—H44A	0.9500
C3—C4	1.394 (11)	C45—C46	1.379 (10)
C3—H3A	0.9900	C45—H45A	0.9500
C3—H3B	0.9900	C46—C47	1.380 (10)
C4—H4A	0.9900	C46—H46A	0.9500
C4—H4B	0.9900	C47—C48	1.390 (10)
C5—H5A	0.9900	C47—H47A	0.9500
C5—H5B	0.9900	C48—H48A	0.9500
C6—C7	1.394 (10)	C49—C54	1.389 (9)
C6—C11	1.398 (9)	C49—C50	1.400 (9)
C7—C8	1.383 (10)	C50—C51	1.387 (9)
C7—H7A	0.9500	C50—H50A	0.9500
C8—C9	1.377 (11)	C51—C52	1.366 (11)
C8—H8A	0.9500	C51—H51A	0.9500
C9—C10	1.373 (11)	C52—C53	1.386 (11)
C9—H9A	0.9500	C52—H52A	0.9500
C10—C11	1.390 (10)	C53—C54	1.398 (10)
C10—H10A	0.9500	C53—H53A	0.9500
C11—H11A	0.9500	C54—H54A	0.9500
C12—C13	1.385 (10)	C1TA—C6TA	1.367 (13)
C12—C17	1.396 (9)	C1TA—C2TA	1.374 (14)
C13—C14	1.389 (10)	C1TA—C7TA	1.522 (12)
C13—H13A	0.9500	C2TA—C3TA	1.391 (14)
C14—C15	1.374 (11)	C2TA—H2TA	0.9500
C14—H14A	0.9500	C3TA—C4TA	1.358 (16)
C15—C16	1.382 (11)	C3TA—H3TA	0.9500
C15—H15A	0.9500	C4TA—C5TA	1.378 (15)
C16—C17	1.396 (9)	C4TA—H4TA	0.9500
C16—H16A	0.9500	C5TA—C6TA	1.383 (13)
C17—H17A	0.9500	C5TA—H5TA	0.9500
C18—C19	1.400 (10)	C6TA—H6TA	0.9500
C18—C23	1.404 (9)	C7TA—H7TA	0.9800
C19—C20	1.384 (10)	C7TA—H7TB	0.9800
C19—H19A	0.9500	C7TA—H7TC	0.9800
C20—C21	1.384 (12)	C1TB—C2TB	1.36 (2)
C20—H20A	0.9500	C1TB—C6TB	1.41 (2)
C21—C22	1.388 (12)	C1TB—H1TA	0.9500
C21—H21A	0.9500	C2TB—C3TB	1.34 (2)
C22—C23	1.388 (10)	C2TB—H2TB	0.9500
C22—H22A	0.9500	C3TB—C4TB	1.354 (18)
C23—H23A	0.9500	C3TB—H3TB	0.9500
C24—C25	1.378 (10)	C4TB—C5TB	1.305 (18)
C24—C29	1.404 (10)	C4TB—C7TC	1.44 (3)
C25—C26	1.399 (11)	C4TB—H4TB	0.9500
C25—H25A	0.9500	C5TB—C6TB	1.368 (17)
C26—C27	1.377 (12)	C5TB—H5TB	0.9500
C26—H26A	0.9500	C6TB—C7TB	1.35 (2)
C27—C28	1.372 (12)	C6TB—H6TB	0.9500
C27—H27A	0.9500	C7TB—H7TD	0.9800
C28—C29	1.395 (10)	C7TB—H7TE	0.9800
C28—H28A	0.9500	C7TB—H7TF	0.9800
C29—H29A	0.9500	C7TC—H7TG	0.9800
C30—H30A	0.9900	C7TC—H7TH	0.9800
C30—H30B	0.9900	C7TC—H7TI	0.9800
C31—C36	1.400 (9)		
			
C2—Ni1—C1	42.5 (3)	P3—C30—P4	115.3 (3)
C2—Ni1—P1	141.0 (2)	P3—C30—H30A	108.4
C1—Ni1—P1	98.9 (2)	P4—C30—H30A	108.4
C2—Ni1—P4	107.9 (2)	P3—C30—H30B	108.4
C1—Ni1—P4	149.8 (2)	P4—C30—H30B	108.4
P1—Ni1—P4	109.21 (7)	H30A—C30—H30B	107.5
C4—Ni2—C3	41.4 (3)	C36—C31—C32	115.9 (6)
C4—Ni2—P2	106.9 (2)	C36—C31—P3	118.0 (5)
C3—Ni2—P2	148.3 (2)	C32—C31—P3	126.0 (5)
C4—Ni2—P3	144.8 (2)	C31—C32—C33	122.5 (7)
C3—Ni2—P3	104.2 (2)	C31—C32—H32A	118.7
P2—Ni2—P3	106.67 (7)	C33—C32—H32A	118.7
C6—P1—C12	101.5 (3)	C34—C33—C32	119.4 (8)
C6—P1—C5	100.9 (3)	C34—C33—H33A	120.3
C12—P1—C5	102.4 (3)	C32—C33—H33A	120.3
C6—P1—Ni1	107.00 (19)	C35—C34—C33	119.1 (7)
C12—P1—Ni1	119.8 (2)	C35—C34—H34A	120.5
C5—P1—Ni1	121.9 (2)	C33—C34—H34A	120.5
C18—P2—C24	99.5 (3)	C34—C35—C36	121.8 (7)
C18—P2—C5	103.0 (3)	C34—C35—H35A	119.1
C24—P2—C5	99.9 (3)	C36—C35—H35A	119.1
C18—P2—Ni2	111.1 (2)	C35—C36—C31	121.2 (7)
C24—P2—Ni2	121.3 (2)	C35—C36—H36A	119.4
C5—P2—Ni2	118.9 (2)	C31—C36—H36A	119.4
C31—P3—C37	101.4 (3)	C38—C37—C42	118.7 (7)
C31—P3—C30	100.5 (3)	C38—C37—P3	123.7 (5)
C37—P3—C30	103.7 (3)	C42—C37—P3	117.6 (5)
C31—P3—Ni2	111.9 (2)	C37—C38—C39	121.2 (7)
C37—P3—Ni2	118.9 (2)	C37—C38—H38A	119.4
C30—P3—Ni2	117.8 (2)	C39—C38—H38A	119.4
C49—P4—C43	100.1 (3)	C38—C39—C40	119.8 (7)
C49—P4—C30	101.5 (3)	C38—C39—H39A	120.1
C43—P4—C30	98.9 (3)	C40—C39—H39A	120.1
C49—P4—Ni1	109.9 (2)	C41—C40—C39	120.1 (7)
C43—P4—Ni1	122.8 (2)	C41—C40—H40A	120.0
C30—P4—Ni1	120.1 (2)	C39—C40—H40A	120.0
C2—C1—Ni1	68.8 (4)	C40—C41—C42	119.8 (7)
C2—C1—H1A	116.8	C40—C41—H41A	120.1
Ni1—C1—H1A	116.8	C42—C41—H41A	120.1
C2—C1—H1B	116.8	C41—C42—C37	120.4 (7)
Ni1—C1—H1B	116.8	C41—C42—H42A	119.8
H1A—C1—H1B	113.8	C37—C42—H42A	119.8
C1—C2—Ni1	68.7 (4)	C44—C43—C48	118.5 (6)
C1—C2—H2A	116.8	C44—C43—P4	121.1 (5)
Ni1—C2—H2A	116.8	C48—C43—P4	120.4 (5)
C1—C2—H2B	116.8	C43—C44—C45	121.1 (6)
Ni1—C2—H2B	116.8	C43—C44—H44A	119.4
H2A—C2—H2B	113.8	C45—C44—H44A	119.4
C4—C3—Ni2	69.1 (4)	C46—C45—C44	119.8 (7)
C4—C3—H3A	116.7	C46—C45—H45A	120.1
Ni2—C3—H3A	116.7	C44—C45—H45A	120.1
C4—C3—H3B	116.7	C45—C46—C47	120.0 (7)
Ni2—C3—H3B	116.7	C45—C46—H46A	120.0
H3A—C3—H3B	113.8	C47—C46—H46A	120.0
C3—C4—Ni2	69.5 (4)	C46—C47—C48	120.2 (7)
C3—C4—H4A	116.7	C46—C47—H47A	119.9
Ni2—C4—H4A	116.7	C48—C47—H47A	119.9
C3—C4—H4B	116.7	C47—C48—C43	120.4 (7)
Ni2—C4—H4B	116.7	C47—C48—H48A	119.8
H4A—C4—H4B	113.7	C43—C48—H48A	119.8
P2—C5—P1	115.5 (3)	C54—C49—C50	118.0 (6)
P2—C5—H5A	108.4	C54—C49—P4	126.4 (5)
P1—C5—H5A	108.4	C50—C49—P4	115.6 (5)
P2—C5—H5B	108.4	C51—C50—C49	121.1 (7)
P1—C5—H5B	108.4	C51—C50—H50A	119.5
H5A—C5—H5B	107.5	C49—C50—H50A	119.5
C7—C6—C11	117.1 (6)	C52—C51—C50	120.4 (7)
C7—C6—P1	122.8 (5)	C52—C51—H51A	119.8
C11—C6—P1	120.0 (5)	C50—C51—H51A	119.8
C8—C7—C6	121.3 (7)	C51—C52—C53	119.8 (7)
C8—C7—H7A	119.3	C51—C52—H52A	120.1
C6—C7—H7A	119.3	C53—C52—H52A	120.1
C9—C8—C7	120.6 (7)	C52—C53—C54	120.2 (7)
C9—C8—H8A	119.7	C52—C53—H53A	119.9
C7—C8—H8A	119.7	C54—C53—H53A	119.9
C8—C9—C10	119.4 (7)	C49—C54—C53	120.5 (6)
C8—C9—H9A	120.3	C49—C54—H54A	119.7
C10—C9—H9A	120.3	C53—C54—H54A	119.7
C9—C10—C11	120.3 (7)	C6TA—C1TA—C2TA	118.1 (9)
C9—C10—H10A	119.9	C6TA—C1TA—C7TA	120.9 (9)
C11—C10—H10A	119.8	C2TA—C1TA—C7TA	120.9 (9)
C10—C11—C6	121.3 (7)	C1TA—C2TA—C3TA	120.8 (11)
C10—C11—H11A	119.4	C1TA—C2TA—H2TA	119.6
C6—C11—H11A	119.4	C3TA—C2TA—H2TA	119.6
C13—C12—C17	119.5 (6)	C4TA—C3TA—C2TA	119.8 (11)
C13—C12—P1	123.0 (5)	C4TA—C3TA—H3TA	120.1
C17—C12—P1	117.6 (5)	C2TA—C3TA—H3TA	120.1
C12—C13—C14	120.5 (7)	C3TA—C4TA—C5TA	120.7 (10)
C12—C13—H13A	119.8	C3TA—C4TA—H4TA	119.7
C14—C13—H13A	119.8	C5TA—C4TA—H4TA	119.7
C15—C14—C13	120.0 (7)	C6TA—C5TA—C4TA	118.5 (10)
C15—C14—H14A	120.0	C6TA—C5TA—H5TA	120.8
C13—C14—H14A	120.0	C4TA—C5TA—H5TA	120.8
C16—C15—C14	120.3 (7)	C1TA—C6TA—C5TA	122.2 (10)
C16—C15—H15A	119.8	C1TA—C6TA—H6TA	118.9
C14—C15—H15A	119.8	C5TA—C6TA—H6TA	118.9
C15—C16—C17	120.1 (7)	C1TA—C7TA—H7TA	109.5
C15—C16—H16A	119.9	C1TA—C7TA—H7TB	109.5
C17—C16—H16A	119.9	H7TA—C7TA—H7TB	109.5
C16—C17—C12	119.6 (7)	C1TA—C7TA—H7TC	109.5
C16—C17—H17A	120.2	H7TA—C7TA—H7TC	109.5
C12—C17—H17A	120.2	H7TB—C7TA—H7TC	109.5
C19—C18—C23	117.1 (6)	C2TB—C1TB—C6TB	117.4 (14)
C19—C18—P2	125.2 (5)	C2TB—C1TB—H1TA	121.3
C23—C18—P2	117.6 (5)	C6TB—C1TB—H1TA	121.3
C20—C19—C18	120.7 (7)	C3TB—C2TB—C1TB	118.9 (15)
C20—C19—H19A	119.6	C3TB—C2TB—H2TB	120.5
C18—C19—H19A	119.6	C1TB—C2TB—H2TB	120.5
C19—C20—C21	121.1 (8)	C2TB—C3TB—C4TB	124.0 (16)
C19—C20—H20A	119.4	C2TB—C3TB—H3TB	118.0
C21—C20—H20A	119.4	C4TB—C3TB—H3TB	118.0
C22—C21—C20	119.6 (8)	C5TB—C4TB—C3TB	118.0 (14)
C22—C21—H21A	120.2	C5TB—C4TB—C7TC	108.6 (18)
C20—C21—H21A	120.2	C3TB—C4TB—C7TC	133 (2)
C23—C22—C21	119.1 (7)	C5TB—C4TB—H4TB	121.0
C23—C22—H22A	120.4	C3TB—C4TB—H4TB	121.0
C21—C22—H22A	120.4	C7TC—C4TB—H4TB	12.5
C22—C23—C18	122.4 (7)	C4TB—C5TB—C6TB	121.8 (13)
C22—C23—H23A	118.8	C4TB—C5TB—H5TB	119.1
C18—C23—H23A	118.8	C6TB—C5TB—H5TB	119.1
C25—C24—C29	118.3 (7)	C7TB—C6TB—C5TB	122.9 (17)
C25—C24—P2	122.9 (6)	C7TB—C6TB—C1TB	117.5 (18)
C29—C24—P2	118.8 (5)	C5TB—C6TB—C1TB	119.6 (14)
C24—C25—C26	121.2 (8)	C7TB—C6TB—H6TB	3.2
C24—C25—H25A	119.4	C5TB—C6TB—H6TB	120.2
C26—C25—H25A	119.4	C1TB—C6TB—H6TB	120.2
C27—C26—C25	119.8 (8)	C6TB—C7TB—H7TD	109.5
C27—C26—H26A	120.1	C6TB—C7TB—H7TE	109.5
C25—C26—H26A	120.1	H7TD—C7TB—H7TE	109.5
C26—C27—C28	120.0 (7)	C6TB—C7TB—H7TF	109.5
C26—C27—H27A	120.0	H7TD—C7TB—H7TF	109.5
C28—C27—H27A	120.0	H7TE—C7TB—H7TF	109.5
C27—C28—C29	120.5 (8)	C4TB—C7TC—H7TG	109.5
C27—C28—H28A	119.7	C4TB—C7TC—H7TH	109.5
C29—C28—H28A	119.7	H7TG—C7TC—H7TH	109.5
C28—C29—C24	120.2 (7)	C4TB—C7TC—H7TI	109.5
C28—C29—H29A	119.9	H7TG—C7TC—H7TI	109.5
C24—C29—H29A	119.9	H7TH—C7TC—H7TI	109.5
